# Contrasting consequences of podocyte insulin-like growth factor 1 receptor inhibition

**DOI:** 10.1016/j.isci.2024.109749

**Published:** 2024-04-16

**Authors:** Jennifer A. Hurcombe, Fern Barrington, Micol Marchetti, Virginie M.S. Betin, Emily E. Bowen, Abigail C. Lay, Lan Ni, Lusyan Dayalan, Robert J.P. Pope, Paul T. Brinkkoetter, Martin Holzenberger, Gavin I. Welsh, Richard J.M. Coward

**Affiliations:** 1 Bristol Renal, University of Bristol, Bristol, UK; 2 Department II of Internal Medicine and Center for Molecular Medicine Cologne (CMMC), Faculty of Medicine and University Hospital Cologne, University of Cologne, Cologne, Germany; 3 Sorbonne University, INSERM, Paris, France

**Keywords:** biological sciences, Molecular biology, cell biology

## Abstract

Insulin signaling to the glomerular podocyte via the insulin receptor (IR) is critical for kidney function. In this study we show that near-complete knockout of the closely related insulin-like growth factor 1 receptor (IGF1R) in podocytes is detrimental, resulting in albuminuria *in vivo* and podocyte cell death *in vitro*. In contrast, partial podocyte IGF1R knockdown confers protection against doxorubicin-induced podocyte injury. Proteomic analysis of cultured podocytes revealed that while near-complete loss of podocyte IGF1R results in the downregulation of mitochondrial respiratory complex I and DNA damage repair proteins, partial IGF1R inhibition promotes respiratory complex expression. This suggests that altered mitochondrial function and resistance to podocyte stress depends on the level of IGF1R suppression, the latter determining whether receptor inhibition is protective or detrimental. Our work suggests that the partial suppression of podocyte IGF1R could have therapeutic benefits in treating albuminuric kidney disease.

## Highlights

• Near-complete loss of podocyte IGF1R is highly detrimental
• Near-complete loss of podocyte IGF1R results in mitochondrial dysfunction
• Partial podocyte IGF1R inhibition is beneficial in protecting against oxidative stress

## Graphical abstract

**Figure:**
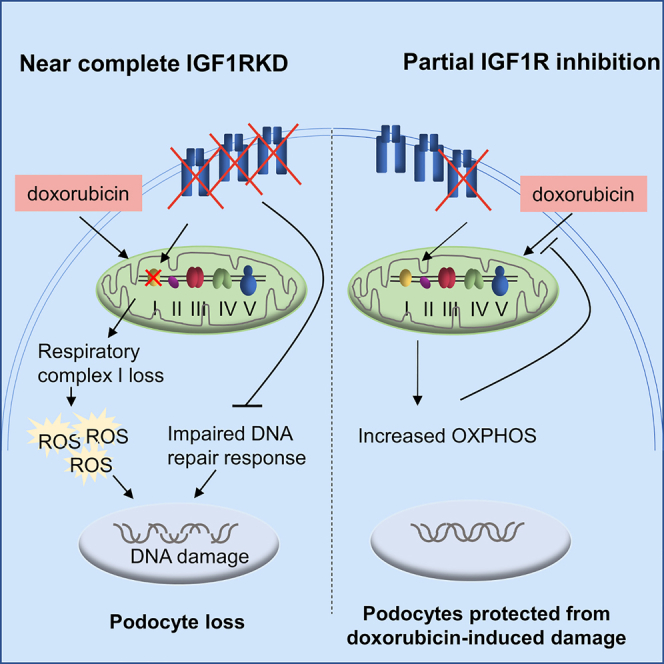

## Introduction

Podocytes are key cells in maintaining the integrity of the glomerular filtration barrier of the kidney. Their loss induces the development of albuminuria, which is an independent risk factor for end-stage-renal-failure^1^ and cardiovascular morbidity.^2^ Therefore, identifying signaling pathways affecting podocyte homeostasis and developing strategies to ameliorate the effects of cellular stress in podocytes is important.

Insulin and insulin-like growth factor (IGF) signaling control many aspects of metabolism, growth, and survival in biological systems.^3^ These ligands and the receptors through which they signal [the insulin receptor (IR) and insulin-like growth factor 1 receptor (IGF1R)] exhibit significant structural similarities allowing crosstalk between them. IGF1 is capable of binding to the IR and insulin to the IGF1R, albeit with much lower affinity than to their cognate receptors.^4^^,^^5^ However, their functions are not interchangeable: constitutive IR knockout mice develop early postnatal diabetes and die of ketoacidosis after several days,^6^ while mice lacking the IGF1R exhibit severe growth retardation along with lethal respiratory failure at birth.^7^^,^^8^ Numerous cell type-specific IGF1R knockout mouse models have been generated and show that while IGF1R activity is critical for the development and function of some cell types,^9^^,^^10^ in others, this knockout does not adversely affect the phenotype.^10^^,^^11^ It is also clear that genetic knockout of the IGF1R can have cell type-dependent metabolic effects,^10^^,^^12^^,^^13^ challenging the conventional view that IGF1 signaling is only associated with growth, differentiation and anti-apoptotic effects and insulin signaling primarily involved in metabolic processes.

We,^14^^,^^15^^,^^16^ and others^17^ established that insulin signaling to the podocyte via the IR is critical for podocyte function. Moreover, there is evidence that IGF signaling can also directly affect podocyte biology resulting in both deleterious and beneficial effects.^16^^,^^18^^,^^19^^,^^20^ Our previous work showed that knockdown of the IGF1R *in vitro* in human podocytes by 99% is highly detrimental resulting in cell death, suggesting that IGF signaling is a critical survival pathway in this cell type.^20^ Furthermore, IGF signaling inhibits podocyte apoptosis^19^ and transgenic mice expressing podocyte-specific dominant negative IGF1R have abnormal, small glomeruli with evidence of podocyte foot process effacement.^18^ Conversely, the inhibition of insulin/IGF signaling was found to be protective in a mouse model of podocyte mitochondrial dysfunction.^16^ However, the mechanisms responsible for these differential effects of IGF signaling in the podocyte are unknown.

In this study, we show that near total loss of murine podocyte IGF1R is detrimental, while partial inhibition provides protection from doxorubicin-induced podocyte injury. We also present proteomic analysis identifying potential mechanisms underlying these seemingly contradictory effects.

## Results

### Podocyte partial insulin-like growth factor 1 receptor knockdown mice have no apparent basal renal phenotype but are protected from doxorubicin-induced nephropathy

**Figure 1:**
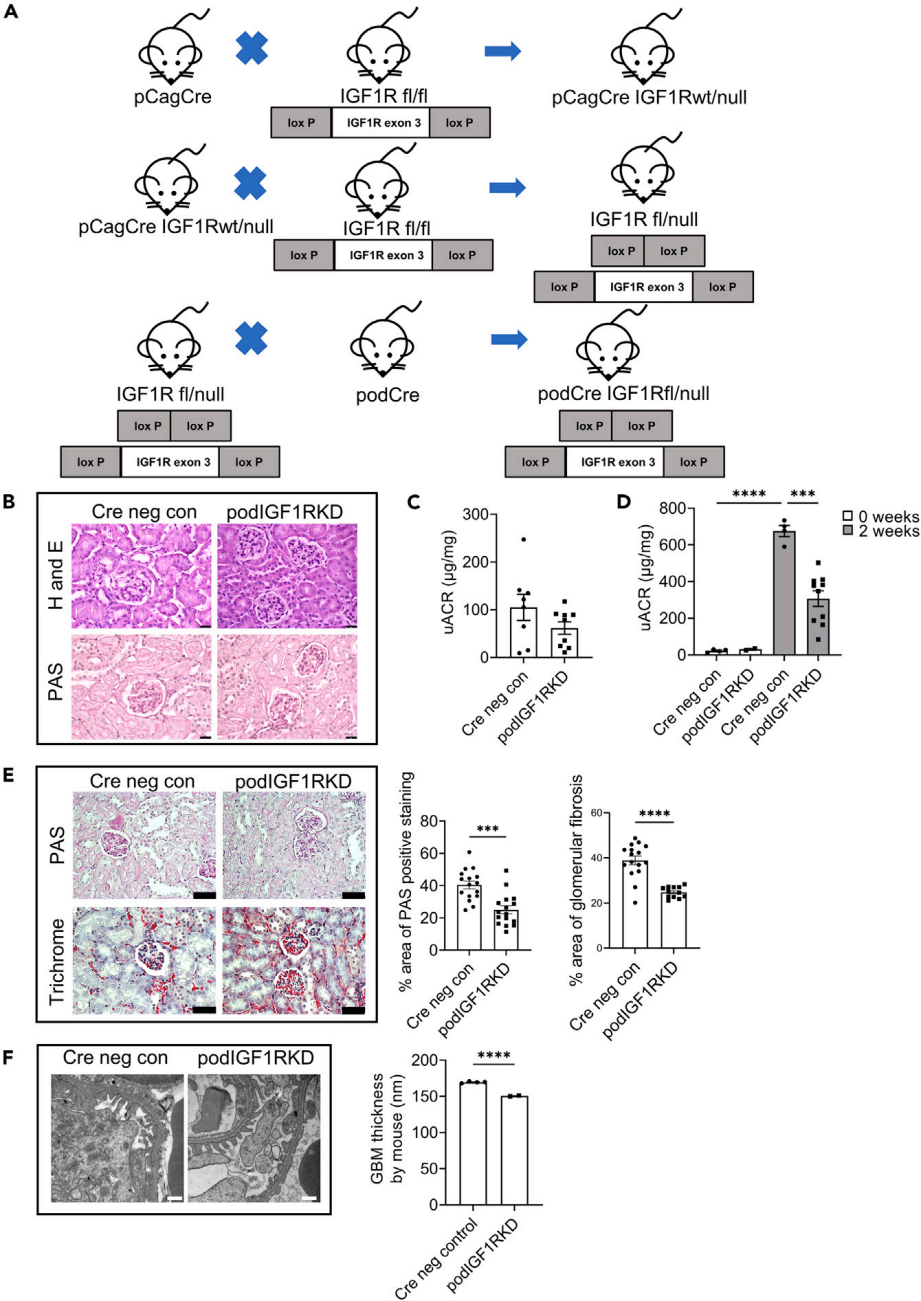
Podocyte partial IGF1RKD knockdown mice have no apparent basal renal phenotype but are protected from doxorubicin-induced nephropathy (A) Breeding scheme used to generate podIGF1RKD mouse model. (B) Haematoxylin and eosin (H&E) and periodic acid-Schiff (PAS) staining. No apparent change in the histological appearance of podIGF1RKD mice at 9 months. Scale bar = 25 μm. (C) Urine albumin: creatinine (uACR) of podIGF1RKD at 9 months. Data are represented as the mean ± SEM, n = 8–9 animals per group. (D) uACR in doxorubicin-treated mice is ∼50% lower in podIGF1RKD mice relative to littermate controls at 2 weeks. Data are represented as the mean ± SEM, one-way ANOVA with Tukey’s multiple comparison test, ∗∗∗∗*p* < 0.0001, ∗∗∗*p* < 0.001, n = 3–9 mice per group. (E) Representative PAS and Masson’s trichrome staining in kidney sections from doxorubicin-treated podIGF1RKD and Cre-negative littermate control mice. Scale bar = 25 μm. Quantification was performed by measuring the area of glomerular PAS-positive staining or blue coloration (indicating fibrosis in Masson’s trichrome staining) in ≥5 glomeruli in 3 mice per group. Data are presented as the mean ± SEM, t test ∗∗∗*p* < 0.001, ∗∗∗∗*p* < 0.0001. (F) Transmission electron microscopy (TEM) shows less glomerular basement membrane (GBM) thickening in doxorubicin challenged podIGF1RKD mice relative to Cre-negative littermate controls. Scale bar = 500 nm. Quantification was performed by measuring ≥20 regions of GBM in ≥2 glomeruli from 3 mice per group. Data expressed as the mean ± SEM, t-test, ∗∗∗∗*p* < 0.0001. See also Figures S1 and S2.

To study the effects of IGF1R depletion in podocytes, transgenic mice were first generated by crossing IGF1R^fl/fl^ mice with mice expressing conventional Cre recombinase under the control of a podocin promotor. However, these mice had no apparent phenotype (Figure S1) and we surmised that this may be due to inefficient Cre-mediated receptor excision. To increase the level of receptor knockdown, one IGF1R allele was made null by initially crossing IGF1R^fl/fl^ mice with mice expressing Cre recombinase downstream of a CAG promotor, a strategy previously used to enhance podocyte *Nck* gene knockout.^21^ Progeny of these mice was then crossed with podocin Cre transgenic mice to generate IGF1R knockdown mice. These mice had one allele that was constitutively null for the IGF1R and one IGF1R allele floxed that could be excised by Cre recombinase (Figure 1A). As with the initial podocin Cre IGF1R^fl/fl^ transgenic mouse model, these podIGF1RKD mice had no evidence of renal damage histologically (Figure 1B). Furthermore, they had a slightly reduced (although not statistically significant) baseline urinary albumin:creatinine (uACR) (Figure 1C) when aged to 9 months, in comparison to wild-type littermate controls.

To investigate whether an additional renal stressor might be necessary to elicit an adverse phenotype, podIGF1RKD mice were given an intravenous tail injection of doxorubicin to induce nephropathy and podocyte injury. Surprisingly, rather than exacerbating albuminuria, IGF1R knockdown mice were substantially protected from doxorubicin-induced disease progression. Two weeks after doxorubicin administration, uACR was 50% lower (*p* < 0.001) in podIGF1RKD mice compared to IGF1R sufficient littermate controls (Figure 1D). Histological analysis revealed reduced periodic acid-Schiff-positive staining in the glomeruli of doxorubicin-challenged podIGF1RKD mice compared with controls, along with lower levels of collagen deposition, as determined by Masson’s trichrome staining (Figure 1E). Furthermore, less glomerular basement membrane thickening was evident in doxorubicin-challenged podIGF1RKD mice on ultrastructural examination (Figure 1F).

### Pod2.IGF1RKD mice have mildly increased albuminuria at 6 months

**Figure 2:**
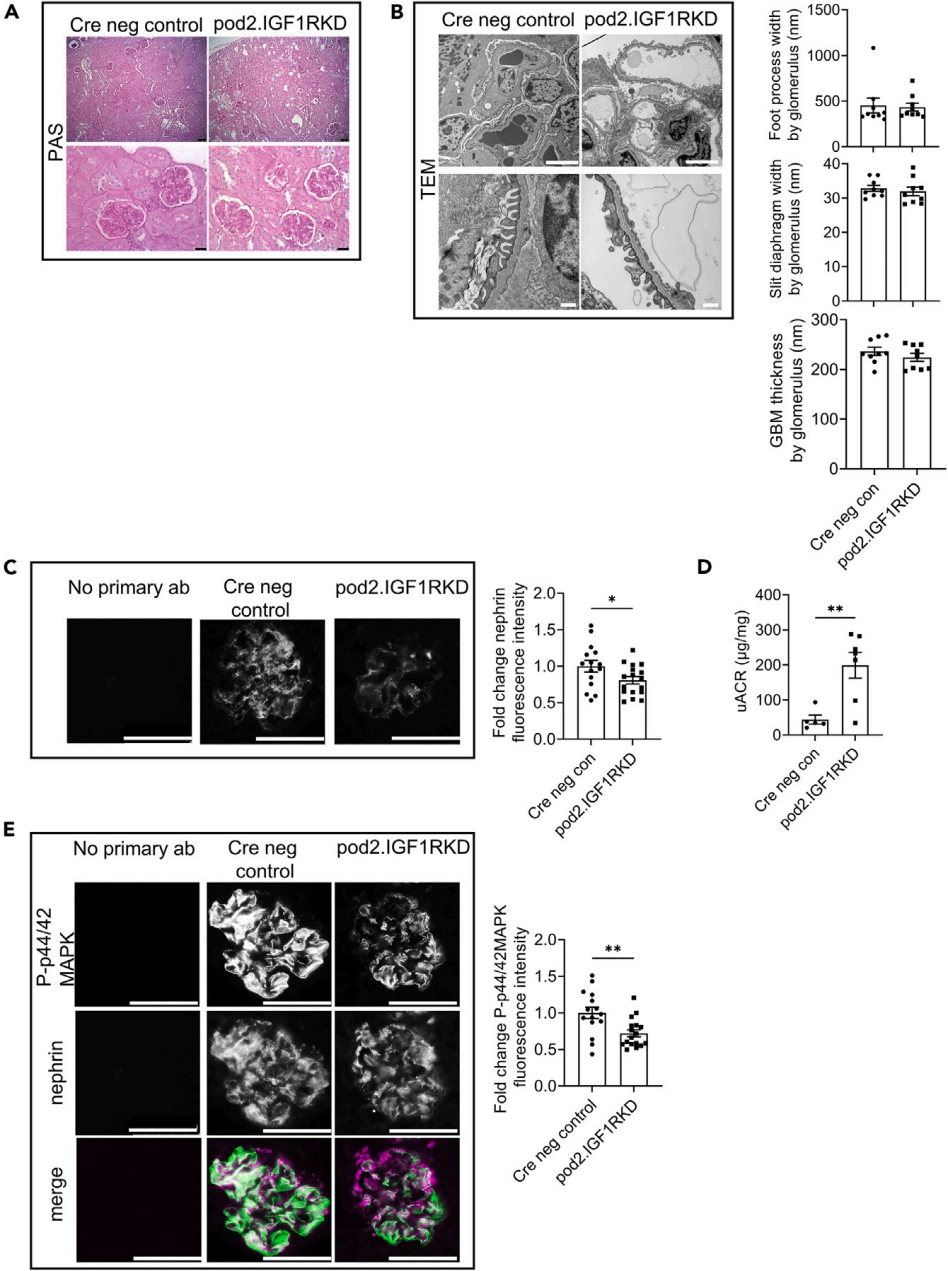
Pod2.IGF1RKD mice have mildly increased albuminuria at 6 months (A) PAS staining; no apparent change in histological appearance of pod2.IGF1RKD mice compared with littermate controls. Scale bar upper panel = 75 μm, lower panel = 25 μm. (B) Transmission electron microscopy (TEM); no apparent change in ultrastructural appearance in pod2IGF1RKD mice. Scale bar upper panel = 5 μm, lower panel = 500 nm. No differences in slit diaphragm width, foot process width or glomerular basement membrane thickness were observed. Data are represented as the mean ± SEM. (C) Immunofluorescence analysis of kidney sections shows reduced levels of nephrin in pod2.IGF1RKD relative to control mice. Quantification was performed by measuring glomerular fluorescence intensity in ≥5 glomeruli in 3 mice per group expressed as the mean ± SEM, t-test, ∗*p* < 0.05. (D) uACR of pod2.IGF1RKO and littermate control mice at 24 weeks. Data are expressed as the mean ± SEM, t-test, ∗∗*p* < 0.005. n = 5–7. (E) Immunofluorescence analysis of kidney sections showing reduced levels and reduced colocalization of P-p44/42 MAPK and nephrin in pod2.IGF1RKD relative to control mice. Scale bar = 50 μm. Quantification was performed by measuring glomerular fluorescence intensity expressed as the mean ± SEM in ≥5 glomeruli in 3 mice per group. t-test, ∗∗*p* < 0.005. See also Figures S2–S4.

We speculated that the lack of a detrimental basal phenotype in podIGF1RKD mice might still be due to incomplete receptor knockdown. To achieve more efficient gene excision, an additional transgenic mouse model was developed with one allele null for IGF1R and the other controlled by a new podocin Cre driver (pod2.IGF1RKD) that has been shown to increase Cre-mediated podocyte floxed gene excision in mice as it is resistant to epigenetically driven loss of Cre expression.^22^ RNAscope *in situ* hybridization was performed to confirm podocyte IGF1R mRNA knockdown in pod2.IGF1RKD. This demonstrated a >80% knockdown (*p* < 0.01) in the podocytes of new pod2.IGF1RKD mice (Figure S2). While the original podIGF1RKD mice were not optimally fixed for RNAscope, resulting in a weak signal in the glomeruli of these mice, there was evidence of IGF1R expression in some podocytes suggesting higher podocyte receptor expression in comparison to the pod2.IGF1RKD mice (Figure S2). Pod2.IGF1RKD mice did not show any major changes in renal histology (Figure 2A), ultrastructural analysis (Figure 2B), body weight, or blood glucose (Figure S3). However, they did have significantly reduced (*p* < 0.05) levels of nephrin expression (Figure 2C) and significantly increased (*p* < 0.01) uACR levels at 6 months relative to controls (Figure 2D). Cre toxicity in our “mixed” genetic background model was excluded as a driver of albuminuria (Figure S4) as was previously reported for pod2Cre C57/Bl6 mice aged to 12 months.^22^ To determine the effect of podocyte IGF1R loss on basal IGF signaling, immunofluorescent analysis was performed and showed a significant decrease (*p* < 0.01) in podocyte p44/42 MAPK phosphorylation in pod2.IGF1RKD mice (Figure 2E). Due to the apparent contrasting phenotypes with suspected “partial” and “near-complete” loss of IGF1R expression in the podocyte, mouse podocytes were studied *in vitro*, where it was possible to accurately modulate and measure precise IGF1R knockdown levels.

### *In vitro* differential suppression of podocyte insulin-like growth factor 1 receptor activity reveals that partial inhibition is beneficial but near-complete loss is highly detrimental

**Figure 3:**
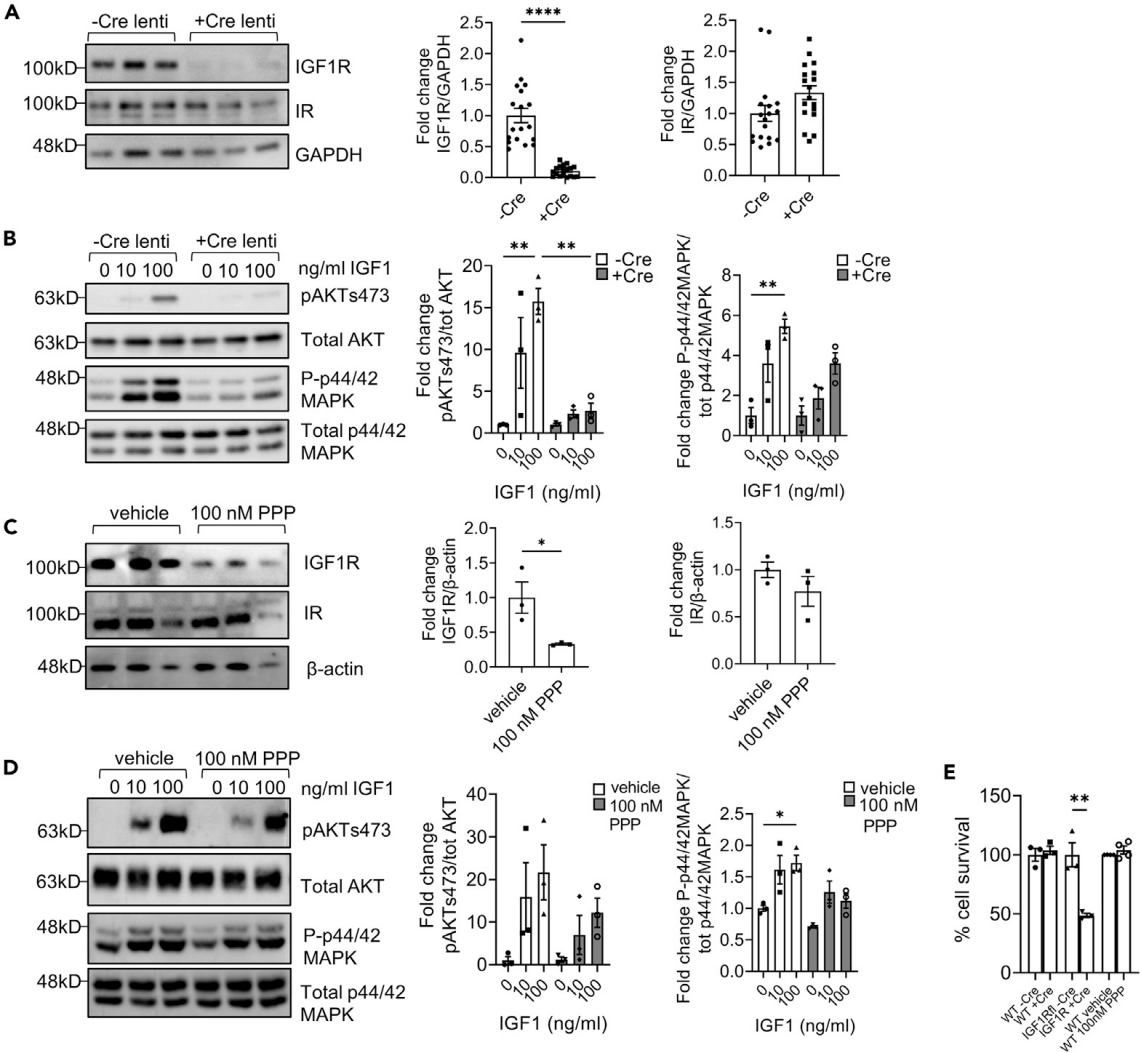
*In vitro* differential suppression of podocyte IGF1R activity reveals that partial inhibition is beneficial but near total loss is highly detrimental (A) Representative Western blot shows >90% reduction of IGF1R protein in NC-IGF1RKD cells but no reduction of IR protein expression. Bar graphs show densitometry expressed as the mean fold change +/− SEM, t-test, ∗∗∗∗*p* < 0.0001, *n* = 18 independent experiments. (B) Phosphorylation of AKT and p44/42MAPK in response to acute IGF1 stimulation at 10 ng and 100 ng/mL for 10 min was significantly reduced in NC-IGF1RKD podocytes. Data are expressed as the mean ± SEM, one-way ANOVA with Tukey’s multiple comparison test, ∗∗*p* < 0.005, *n* = 3 independent experiments. (C) Western blot shows that IGF1R expression is reduced by ∼70% in wild-type podocytes exposed to 100 nM picropodophyllin for 24 h. Data are expressed as the mean ± SEM, t-test, ∗*p* < 0.05, *n* = 3 independent experiments. No significant change in IR expression was observed. (D) Western blot shows the phosphorylation of AKT and p44/42MAPK in response to acute IGF1 stimulation at 10 ng and 100 ng/mL for 10 min in podocytes exposed to 100 nM picropodophyllin for 24 h. Data expressed as the mean ± SEM, ∗*p* < 0.05, *n* = 3 independent experiments. (E) ∼50% of NC-IGF1RKD cells survive 7 days after gene excision. Treatment of wild-type podocytes with 100 nM picropodophyllin for 24 h has no effect on cell survival. Data are expressed as the mean ± SEM, t-test, ∗∗*p* < 0.005, n = 3–4 independent experiments. See also Figures S5 and S6.

To correlate the degree of IGF1R suppression with cellular phenotype and elucidate the mechanistic processes involved, we developed *in vitro* cell culture models of both near-complete and partial receptor suppression. To achieve near-complete knockout of the IGF1R, a conditionally immortalized podocyte cell line was generated from homozygous IGF1R^fl/fl^ mice. Cells were differentiated by thermo-switching from 33°C to 37°C for 7 days before transduction with a Cre expressing lentivirus to genetically excise the receptor *in vitro,* thereby knocking down IGF1R expression (NC-IGF1RKD podocytes) (Figure S5). The level of IGF1R protein in these cells, as shown by Western blotting, was reduced by >90% (*p* < 0.0001) with no effect on IR protein expression (Figure 3A). IGF signaling was suppressed in NC-IGF1RKD podocytes with the phosphorylation of AKT, and p44/42 MAPK in response to IGF1 stimulation significantly (*p* < 0.01) reduced (Figure 3B). To model partial receptor suppression, we treated wild-type differentiated podocytes with the IGF1R-specific inhibitor picropodophyllin.^23^ Picropodophyllin induces the downregulation of the IGF1R in several cell types.^24^^,^^25^ We found that this was also the case in podocytes. A pilot dose-response experiment determined that the optimal dose of picropodophyllin for podocytes, which suppressed IGF1R levels and signaling but showed no cellular toxicity, was 100 nM (Figure S6B). This dose was used for subsequent studies. Treatment of wild-type podocytes with picropodophyllin for 24 h reduced the IGF1R level by ∼70%, (*p* < 0.05) (P-IGF1RKD) compared with vehicle-treated control cells with no effect on IR expression (Figure 3C). This slightly reduced the phosphorylation of AKT and p44/42 MAPK in response to acute IGF1 stimulation, although this reduction was not statistically significant (Figure 3D). A >90% loss of podocyte IGF1R in NC-IGF1RKD podocytes was detrimental causing ∼50% (*p* < 0.01) cell death 7 days after Cre transduction under basal, non-stressed, conditions (Figure 3E). In contrast, the treatment of wild-type podocytes with picropodophyllin (P-IGF1RKD) had no detrimental effect on cell survival (Figure 3E).

### Partial suppression of insulin-like growth factor 1 receptor activity attenuates doxorubicin and other oxidative stress-induced podocyte death

**Figure 4:**
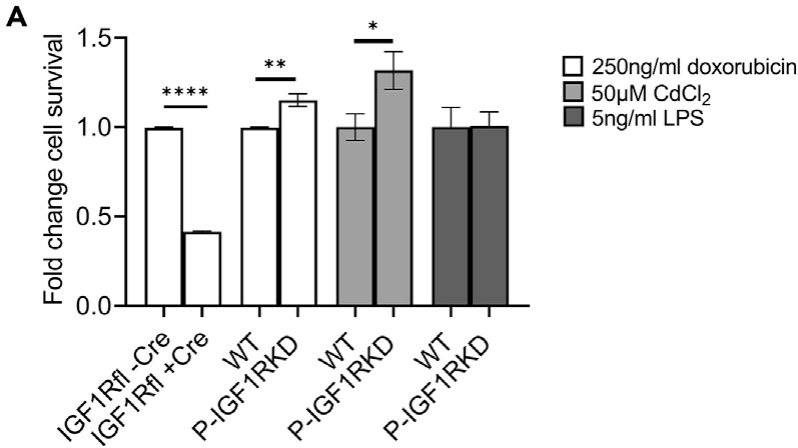
Partial suppression of IGF1R activity attenuates *in vitro* doxorubicin and cadmium chloride -induced podocyte death (A) Fold change in cell survival of NC-IGF1RKO relative to non-transduced control after 24-h exposure to 250 ng/mL doxorubicin and P-IGF1RKD compared to wild-type podocytes after 24-h exposure to 250 ng/mL doxorubicin, 50 μM CdCl_2_ or 5 ng/mL LPS. Data are expressed as mean ± SEM, t-test, ∗*p* < 0.05, ∗∗*p* < 0.005, ∗∗∗∗*p* < 0.0001. n = 3–4 independent experiments. See also Figure S6.

We speculated that the beneficial effect of IGF1R knockdown in doxorubicin-treated podIGF1RKD mice and the contrasting detrimental basal phenotype observed in pod2.IGF1RKD mice might be due to variable efficiency of receptor knockdown and used the *in vitro* models of total and partial IGF1R podocyte suppression to study this. NC-IGF1RKD and P-IGF1RKD picropodophyllin treated podocytes were exposed to 0–1000 ng/mL doxorubicin for 24 h resulting in cell death in a concentration-dependent manner (Figures S6A and S6B). However, while genetic near-complete knockout of the IGF1R had an additional negative effect on cell number (Figures 4 and S6A), doxorubicin-induced cell death was attenuated in P-IGF1RKD cells (Figures 4 and S6B). At a doxorubicin concentration of 250 ng/mL, cell number was ∼60% (*p* < 0.0001) lower in NC-IGF1RKO podocytes compared with non-transduced control cells (Figure 4). In contrast, cell survival was improved by ∼15% (*p* < 0.01) in P-IGF1RKD podocytes relative to wild-type control (Figure 4). To assess if partial podocyte IGF1R suppression may be beneficial in preventing podocyte injury more generally, P-IGF1RKD podocytes were also studied in models of lipopolysaccharide (LPS) and cadmium chloride (CdCl_2_) induced cell death. P-IGF1RKD cells were not protected from LPS-induced death, but cell survival was ∼30% greater (*p* < 0.05) than wild-type controls when exposed to 50 μM CdCl_2_. (Figures 4 and S6C).

### Proteomic analysis of NC-IGF1RKD and P-IGF1RKD podocytes reveals the differential expression of mitochondrial respiratory electron transport chain proteins

**Figure 5:**
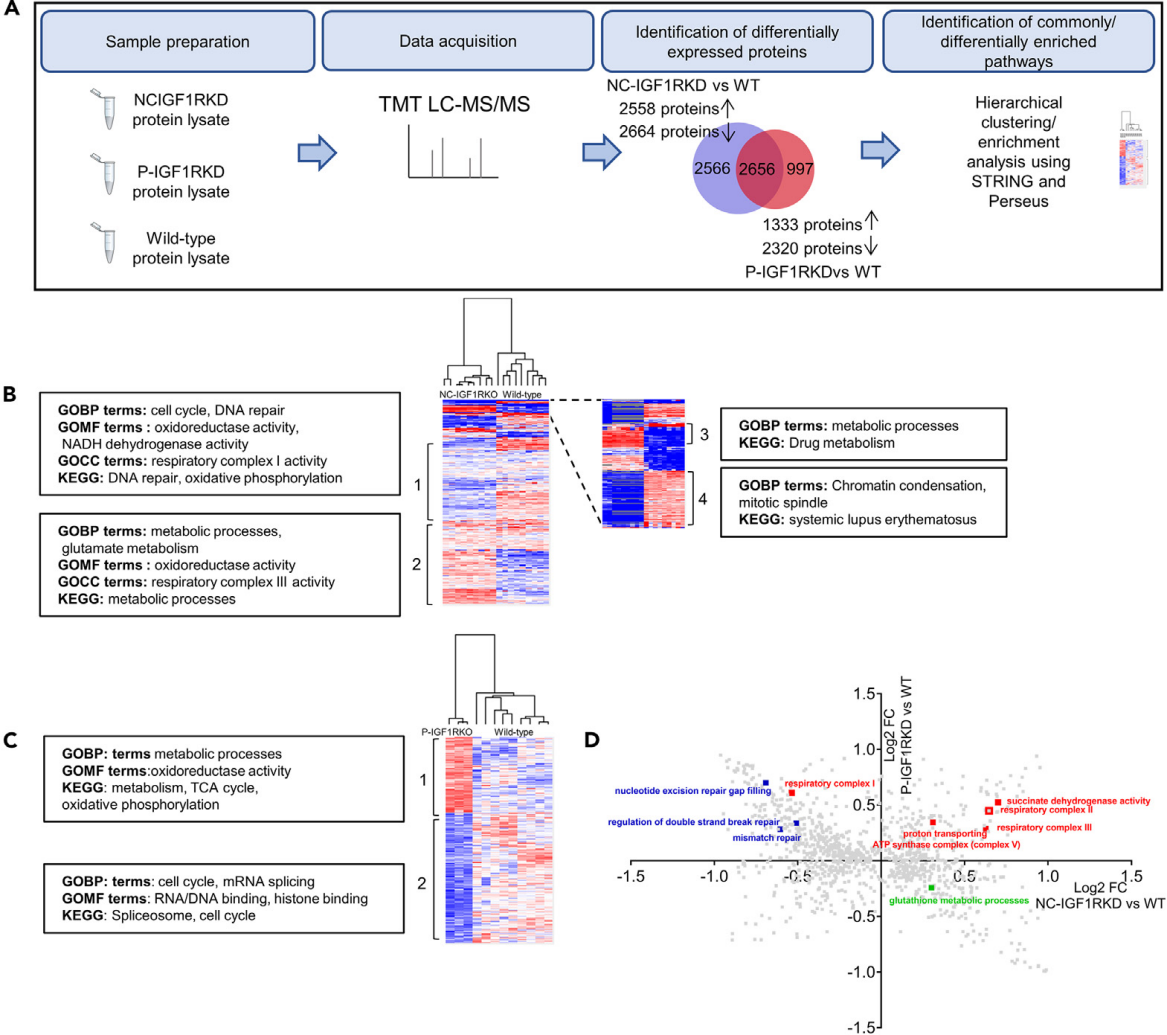
Proteomic analysis of NC-IGF1RKD and P-IGF1RKD podocytes (A) Schematic outlining workflow for proteomic analysis (B) Heatmap shows the hierarchical clustering of NC-IGF1RKD vs. wild-type podocyte proteomes (decreased protein expression in blue, increased expression in red) and the GO and KEGG and terms enriched in four major clusters. (C) Heatmap shows the hierarchical clustering of P-IGF1RKD vs. wild-type podocyte proteomes (decreased protein expression in blue, increased expression in red) and the GO and KEGG and terms enriched in the two major clusters. (D) GO and KEGG 2D enrichment analysis comparing NC-IGF1RKD and P-IGF1RKD proteomes. Selected GO terms associated with mitochondrial function (red), DNA repair (blue), and glutathione metabolism (green) are highlighted. Upregulation of glutathione metabolism and downregulation of DNA repair processes and respiratory complex I is indicated in NC-IGF1RKD but not P-IGF1RKD podocytes. Similar upregulation of respiratory complexes II, III, and V occurs in both datasets. See also Figures S7 and S8.

**Figure 6:**
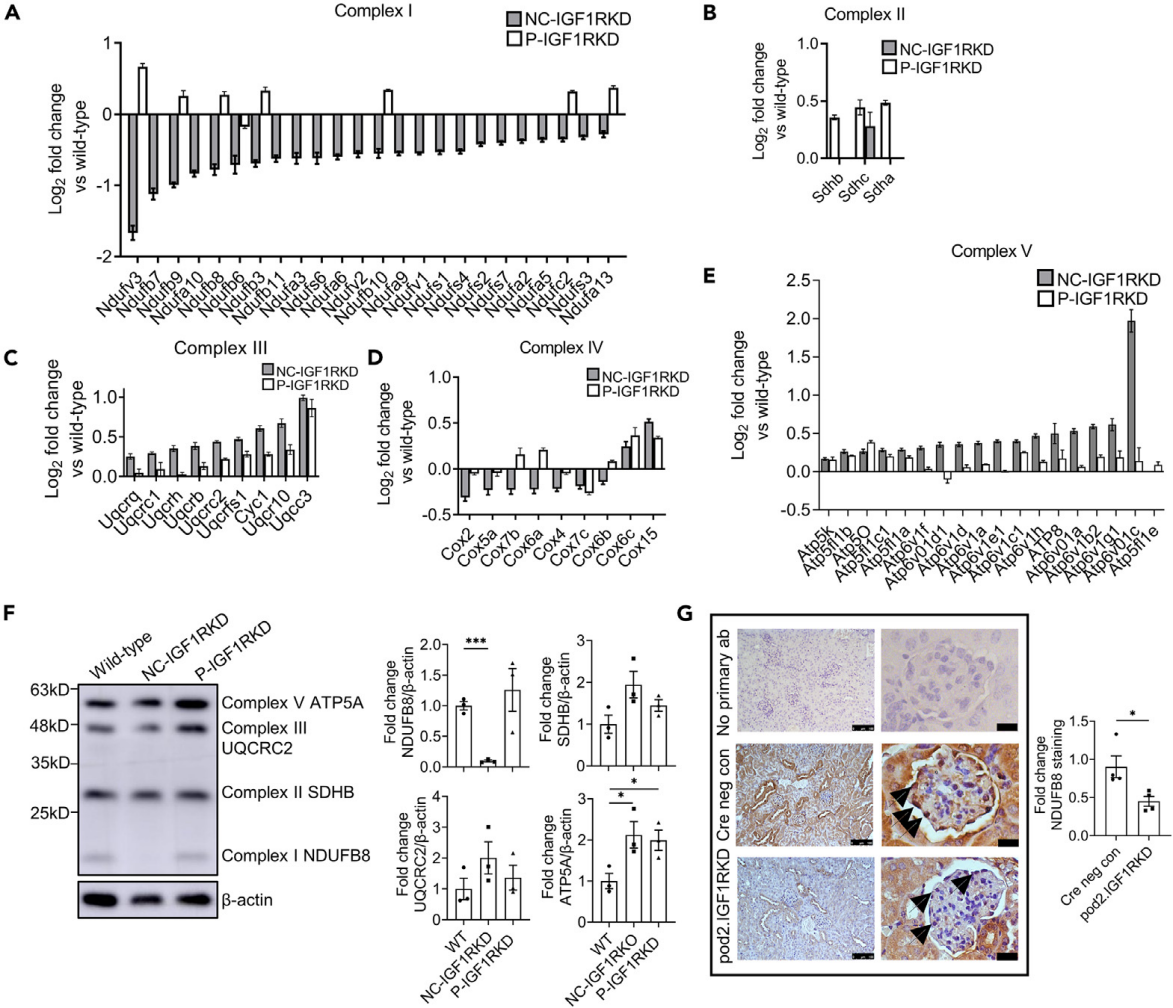
Proteomic analysis of NC-IGF1RKD and P-IGF1RKD podocytes reveals the differential expression of mitochondrial respiratory electron transport chain proteins dependent on the degree of IG1R suppression (A‒E) Graphs showing log_2_ fold change of significantly differentially expressed mitochondrial respiratory complex I (A), complex II (B), complex III (C), complex IV (D), and complex V (E) proteins in NC-IGF1RKD and P-IGF1RKD podocytes compared to wild-type. Data are represented as the mean ± SEM. (F) Representative Western blot and densitometry show reduced expression of the respiratory complex I protein NDUFB8 in NC-IGF1RKD compared with wild-type podocytes but significantly increased the expression of respiratory complex V protein ATP5A in both NC-IGF1RKD and P-IGF1RKD podocytes relative to control. Data are represented as the mean ± SEM, t-test, ∗*p* < 0.05, ∗∗∗*p* < 0.001, *n* = 3 independent experiments. (G) Immunohistochemistry shows reduced podocyte expression (arrowed) of NDUFB8 in pod2.IGF1RKO mice. Scale bar left panel = 100 μm, right panel = 50 μm. Quantification was performed by measuring glomerular signal intensity in 4 glomeruli in 2 mice per group and is expressed as the mean ± SEM, t-test, ∗*p* < 0.05. See also Figure S9.

To identify mechanistic processes responsible for the seemingly contradictory effects of partial and near-complete IGF1R inhibition observed in the *in vivo* and *in vitro* models, tandem mass tagged (TMT) LC MS/MS proteomics was performed to compare the cellular changes that occurred with genetic knockout (NC-IGF1RKD) vs. P-IGF1RKD (100 nM picropodophyllin) in podocytes. Hierarchical clustering and principal component analysis (PCA) confirmed that samples were clustered according to the experimental group (Figures S7A and B). A total of 5222 and 3653 differentially expressed proteins were identified in NC-IGF1RKD and P-IGF1RKD podocytes, respectively, when compared with wild-type cells (Figures 5A and S7C). 2656 proteins were differentially expressed in both NC-IGF1RKD and P-IGF1RKD cells (Figure 5A). We used Perseus software to perform hierarchical clustering and identify GO (Gene Ontology) terms and KEGG (Kyoto Encyclopedia of Genes and Genomes pathways over-represented in the NC-IGF1RKO and P-IGF1RKD proteomic datasets (Figures 5B and 5C; Table S1; Table S2). In both NC-IGF1RKO and P-IGF1RKD podocytes, there was decreased expression of proteins involved in cell cycle progression. NC-IGF1RKD cells also showed the downregulation of DNA damage repair pathways, but the upregulation of glutathione metabolism. STRING (Search Tool for the Retrieval of Interacting Genes/Proteins) enrichment analysis showed similar results (Figure S8). 2D enrichment analysis was performed in Perseus to identify GO terms similarly or differentially enriched in NC-IGF1RKD and P-IGF1RKD podocytes (Figure 5D). Strikingly, components of mitochondrial respiratory complex I were significantly downregulated in NC-IGF1RKD podocytes but showed unchanged or increased expression in the P-IGF1RKD cells (Figures 6A and S9). In contrast, the expression of multiple proteins found in respiratory complexes II, III, and V were significantly increased in both cell types relative to wild-type controls (Figures 6B–6E and S9).

Western blots of NC-IGF1RKD and P-IGF1RKD podocytes were performed to validate the altered expression of mitochondrial respiratory complex proteins indicated by our proteomic analysis. Using the total OXPHOS rodent antibody WB cocktail (Abcam) and comprising antibodies recognizing representative proteins from respiratory complexes I, II, III, and V), expression of the complex I protein NADH dehydrogenase iron-sulphur protein 8 (NDUFB8) was reduced by >90% (*p* < 0.001) in NC-IGF1RKD, but not in P-IGF1RKD podocytes when each was compared with the wild-type control. In contrast, the expression of the complex V protein ATP synthase alpha subunit (ATP5A) was significantly increased (*p* < 0.05) in both NC-IGF1RKD and P-IGF1RKD cells (Figure 6F). In addition, significantly less (*p* < 0.05) NDUFB8 expression occurred *ex vivo* in the podocytes of pod2.IGF1RKD mice compared to littermate controls (Figure 6G).

### Mitochondrial function is impaired in NC-IGF1RKD podocytes

**Figure 7:**
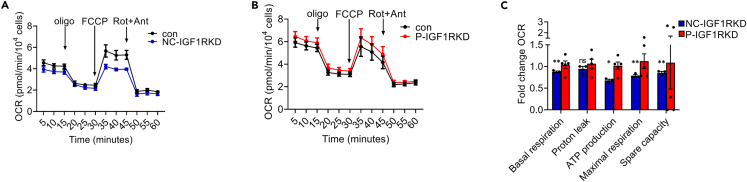
Mitochondrial function is impaired in NC-IGF1RKD podocytes (A) and (B) A Seahorse bioanalyzer was used to measure OCR in NC-IGF1RKD vs. control cells (A) and P-IGF1RKD vs. vehicle-treated podocytes (B), at baseline and following the sequential addition of oligomycin (2 μM), FCCP (1 μM) and rotenone and antimycin A (0.5 μM each) at the times indicated. (C) Basal respiration, ATP production, maximal respiration, and spare capacity are significantly reduced in NC-IGF1RKD podocytes. No significant difference in proton leak was observed. Mitochondrial parameters are expressed as the mean ± SEM fold change in OCR in NC-IGF1RKD vs. control podocytes and P-IGF1RKD vs. vehicle-treated cells. t-test, ∗*p* < 0.05, ∗∗*p* < 0.005, *n* = 3 independent experiments. See also Figure S10.

To investigate whether the reduction in respiratory complex I observed in NC-IGF1RKD podocytes resulted in altered mitochondrial function, the bioenergetic profile of these cells was determined using a flux analyzer to monitor oxygen consumption rates (OCR) (Figure 7A). Cells were imaged following the assay and the GFP signal resulting from Cre expression was used to confirm successful Cre lentiviral transduction (Figure S10). The assay was also performed in P-IGF1RKD podocytes (Figure 7B). NC-IGF1RKD podocytes showed a significant reduction in basal respiration (*p* < 0.01) and total adenosine triphosphate (ATP) synthesis (*p* < 0.05) when compared with control cells but OCR associated with proton leak was unchanged (Figure 7C). Maximal respiration and spare respiratory capacity — defined as the difference between maximal and basal respiration and an indicator of mitochondrial reserve — were also decreased (*p* < 0.01) in NC-IGF1RKD podocytes (Figure 7C). In contrast, P-IGF1RKD podocytes showed a trend (though not statistically significant) toward increases in mitochondrial functional parameters (Figure 7C).

## Discussion

Here we show that podocyte IGF signaling is important for cell survival but that only a fraction of activity is required to maintain function. Moreover, partial suppression of the IGF1R can be protective in conditions of acute cellular stress.

We initially used a transgenic mouse model (podIGF1RKD) to assess podocyte IGF signaling *in vivo*. In light of our previous work showing that near-complete (99%) loss of the IGF1R in cultured human podocytes caused significant cell death,^20^ the lack of a basal renal phenotype in podIGF1RKD mice was unexpected. Furthermore, attempts to elicit a deleterious phenotype by administering doxorubicin, a model of focal segmental glomerulosclerosis (FSGS),^26^ revealed that conversely, these partial podIGF1RKD animals were protected from nephropathy. Although IGF1 signaling has an important pro-survival role, partial loss, or inhibition, of the IGF1R can be beneficial in some contexts. Examples include constitutive heterozygous knockout mice, which have increased life span and protection against oxidative stress^7^^,^^27^ in contrast to IGF1R homozygous knockout that die at birth.^8^ IGF1R heterozygous knockout mice are also protected from colitis and colorectal cancer due to the enhanced preservation of mitochondrial structures and dynamics, alongside increased mitochondrial electron transport chain activation in conditions of oxidative stress.^27^ Neuronal knockout of the IGF1R is protective in mouse models of stroke^28^ and prevents the pathological accumulation of amyloid beta in models of Alzheimer’s disease.^29^^,^^30^^,^^31^^,^^32^ Furthermore, much work has focused on the role of IGF1R activity in tumorigenesis with a view to developing IGF1R inhibitors for therapeutic use in cancer that attenuate the deleterious mitogenic effects of IGF signaling.^33^^,^^34^^,^^35^ Our studies suggest the partial inhibition of IGF1 signaling in the kidney may also be therapeutically beneficial in preventing proteinuric kidney disease progression. This will require further investigation as the *in vitro* experiments showed that picropodophyllin was able to protect against both doxorubicin and CdCl_2_-induced podocyte death (both models of oxidative stress) but did not obviously protect podocytes exposed to LPS which induces pro-inflammatory responses in podocytes.^36^^,^^37^ This suggests that partial IGF1R inhibition is particularly beneficial in the context of oxidative stress. We acknowledge that picropodophyllin has off-target effects. However, it has the advantage that its use has been widely used to partially suppress IGF1R function^23^^,^^38^^,^^39^ and we show that it was able to partially inhibit the IGF1R but not the IR in the podocyte. This is important for our studies as it supports the notion that the level of podocyte IGF1R inhibition required for benefit or harm is crucial in this cell type. Our work suggests approximately 70% receptor knockdown is beneficial in oxidative stress situations, but increased receptor knockdown becomes detrimental. The 82% knockdown of podocyte IGF1R expression in pod2.IGF1RKD mice resulted in significant amounts of albuminuria (suggesting podocyte dysfunction) but was not associated with gross renal histological abnormality. We suspect if we had generated a mouse model with >90% podocyte IGF1R knockdown, (as attained *in vitro* in NC-IGF1RKD podocytes), a more severe renal phenotype would have resulted.

Unbiased analysis of the proteomes of NC-IGF1RKD and P-IGF1RKD podocytes was performed with the aim of identifying altered protein expression and affected pathways that might explain the contrasting observations between near-complete and partial podocyte IGF1R knockdown. The results showed that the inhibition of podocyte IGF1R causes the downregulation of proteins involved in cell cycle progression. This was not unexpected given the role of IGF signaling in growth and proliferation.^40^ The influence of insulin/IGF signaling on podocyte cell cycling is also demonstrated by our recent finding that podocyte knockout of glycogen synthase kinase 3 (GSK3) (downstream of the IR and the IGF1R) causes a severe renal phenotype via cell cycle re-entry and mitotic catastrophe.^41^ Further to this effect of IGF1R deficiency on the cell cycle, there was a notable decrease in the expression of DNA repair proteins, particularly in NC-IGF1RKD podocytes. Reduced expression or inhibition of the IGF1R is associated with defects in DNA repair mechanisms in other settings, sensitizing cells to cytotoxic drugs and radiation treatment in cancer therapy.^42^^,^^43^^,^^44^ Although podocytes are post-mitotic cells with a limited capacity for proliferation, DNA damage responses have a role in maintaining podocyte homeostasis.^45^^,^^46^ It is conceivable that the impairment of this process could have a deleterious impact on podocyte survival.

Proteomic analysis also suggests that near-complete IGF1R loss and partial IGF1R inhibition have differing effects on podocyte mitochondrial function. Podocytes with >90% ablation of the IGF1R showed a marked decrease in the expression of proteins that comprise mitochondrial respiratory complex I but significant upregulation of many components of other respiratory complexes (possibly as an attempt to compensate for complex I loss). Furthermore, analysis of mitochondrial bioenergetics revealed the impairment of mitochondrial function in NC-IGF1RKD podocytes: we observed significantly reduced basal respiration, ATP production, and spare capacity in these cells. Spare respiratory capacity correlates with the ability of mitochondria to respond to increased energy demand in conditions of cellular stress and reduced mitochondrial reserve is associated with many pathological conditions.^47^ The decrease in the bioenergetic capacity of mitochondria in NC-IGF1RKD podocytes occurred in the absence of proton leak, implying that reduced proton production by early oxidative phosphorylation complexes may therefore be responsible for reduced total ATP production in these cells (consistent with the reduction of respiratory complex I shown by the proteomic data). Inhibition of respiratory complex I is known to increase reactive oxygen species (ROS) production^48^^,^^49^ and limit spare respiratory capacity, both of which have been implicated in neuronal damage in Parkinson’s disease.^50^^,^^51^ Furthermore, loss of the IGF1R in astrocytes reduces complex I activity impairing their ability to support neurons in conditions of oxidative stress.^52^ The NC-IGF1RKD proteome revealed increased glutathione metabolism, which is a response to oxidative stress^53^^,^^54^ and suggestive of increased ROS production in these cells. Reduced complex I subunit transcription and mitochondrial respiration have also been reported with muscle-specific combined knockout of the IR and IGF1R.^55^^,^^56^^,^^57^ However, it should be noted that the authors found that muscle-specific IR knockout alone had the same effect, implying that the IR is more important than the IGF1R in regulating mitochondrial function in this cell type.

In contrast, the expression of complex I proteins, in addition to other mitochondrial respiratory complexes, was in many cases increased with the partial pharmacological inhibition of the IGF1R in wild-type podocytes with picropodophyllin. A continuous energy supply mediated by efficient mitochondrial function is important for podocyte homeostasis.^58^ It is therefore conceivable that increased mitochondrial respiratory capacity resulting from partial IGF1R inhibition could have a positive effect on podocyte function when these cells are under stress. Moreover, the upregulation of mitochondrial respiratory chain complexes prevents doxorubicin-induced podocyte apoptosis.^59^ Doxorubicin exerts deleterious effects by reducing mitochondrial respiratory chain function and increasing superoxide production.^60^ This additional insult to NC-IGF1RKD podocytes, causing further impairment to mitochondrial function, could explain the higher degree of cell death that was observed when these cells were exposed to doxorubicin. Conversely, increased oxidative phosphorylation in P-IGF1RKD podocytes may compensate for doxorubicin-induced mitochondrial dysfunction explaining the protective effect of partial IGF1R inhibition in conditions of podocyte stress. However, although the proteomic data convincingly showed the upregulation of many mitochondrial respiratory complex proteins in P-IGF1RKD podocytes, the trend for improved mitochondrial function shown in the Seahorse assay was not statistically significant. It is likely that other mechanistic processes also contribute to the beneficial effects of partial IGF1R inhibition.

In summary, we show that loss of the IGF1R in podocytes affects mitochondrial function, DNA damage responses, and resistance to oxidative stress, dependent on the level of IGF1R suppression. Near-complete loss of podocyte, IGF1R is detrimental showing that IGF1 signaling is important for podocyte function while partial inhibition confers protection from doxorubicin-induced injury. Conceivably, facilitating partial IGF1 signaling in the podocyte could have therapeutic potential in the treatment of kidney diseases, as is currently being assessed in other disease situations.

### Limitations of the study

This study shows that the partial inhibition of podocyte IGF1R is beneficial in models of oxidative stress but not in podocytes challenged with LPS. In order to therapeutically exploit this finding, further investigation will be required to establish whether this protective effect is restricted to conditions of oxidative stress and to precisely determine the optimum level of receptor inhibition.

## STAR★Methods

### Key resources table

**Table:** 

REAGENT or RESOURCE	SOURCE	IDENTIFIER
**Antibodies**		

IRβ	Cell Signaling	Cat# 3025; RRID:AB_2280448
IGF1Rβ	Cell Signaling	Cat# 9750; RRID:AB_10950969
Phospho AKT (ser 473)	Cell Signaling	Cat# 4060; RRID:AB_2315049
AKT	Cell Signaling	Cat# 2920; RRID:AB_1147620
Phospho p44/42 MAPK	Cell Signaling	Cat# 4370; RRID:AB_2315112
P44/42 MAPK	Cell Signaling	Cat# 9102; RRID:AB_330744
NDUFB8	Proteintech	Cat# 14794-1-AP; RRID:AB_2150970
Nephrin	Origene	Cat# BP5030
Anti-WT1 antibody	Merck	Clone 6F-H2 Cat# 05-753
Mouse Anti-beta-Actin Monoclonal Antibody, Unconjugated	Sigma-Aldrich	Clone AC-74 Cat# A5316; RRID:AB_476743
GAPDH	Sigma-Aldrich	Cat# G8795; RRID:AB_1078991
Total OXPHOS Rodent WB antibody cocktail	Abcam	Cat# ab110413; RRID:AB_
Goat anti-Rabbit IgG (H + L) Cross-Adsorbed Secondary Antibody Alexa Fluor® 488	Thermo Fisher Scientific	Cat#A11008; RRID:AB_143165
Goat anti-Guinea-pig IgG (H + L) Cross-Adsorbed Secondary Antibody Alexa Fluor® 568	Thermo Fisher Scientific	Cat# A11075; RRID:AB_ 2534119
Anti-rabbit IgG peroxidase antibody	Sigma-Aldrich	Cat# A6667; RRID:AB_ 258307
Anti-mouse IgG peroxidase antibody	Sigma-Aldrich	Cat# A9044; RRID:AB_ 258431

**Chemicals, peptides, and recombinant proteins**		

Doxorubicin Hydrochloride	Merck	Cat# D1515
Hexadimethrine Bromide	Merck	Cat# H9268
Picropodophyllin	Stratech	Cat# S7668
Buffered neutral formalin	Merck	Cat# HT501128
Histo-Clear II	National Diagnostics	Cat# HS-202
Bovine serum albumin	Merck	Cat# A9647
Protease inhibitor cocktail	Merck	Cat# P8340
Phosphatase inhibitor cocktail 2	Merck	Cat# P5726
Phosphatase inhibitor cocktail 3	Merck	Cat# P0044
Insulin	Biotechne	Cat# 3435
Recombinant Mouse IGF1 protein	Novus	Cat# NBP2-35081
Hematoxylin Solution, Gill No. 1	Sigma	Cat# GHS132
Hoechst 33342	Thermo Fisher Scientific	Cat# H3570

**Critical commercial assays**		

Bethyl mouse albumin ELISA quantitation set	Universal Biologicals	Cat# E90-134
The Creatinine Companion	Exocell	Cat# 1012
Periodic Acid Schiff staining kit	Sigma	Cat# 395B
Trichrome Staining Kit	Sigma	Cat# HT15
RNAScope wash buffer	Biotechne	Cat# 310091
RNAScope target retrieval reagents	Biotechne	Cat# 322000
RNAScope protease plus	Biotechne	Cat# 322330
RNAScope 2.5 HD duplex detection reagents	Biotechne	Cat# 322500
RNAScope probe Mm Igf1r-E3	Biotechne	Cat# 417798
RNAScope probe Mm WT1-C2	Biotechne	Cat# 432711-C2
RIPA buffer	Fisher	Cat# 10017003
Clarity Western ECL substrate	BioRad	Cat# 1705061
SignalStain Boost IHC detection reagent (HRP rabbit)	Cell Signaling	Cat# 8114
SignalStain DAB substrate kit	Cell Signaling	Cat# 8059
Seahorse Xfp cell culture miniplates	Agilent Technologies	Cat# 103025-100
Seahorse XFe96 cell mito stress test kit	Agilent Technologies	Cat# 103015-100

**Experimental models: Cell lines**		

Mouse wild-type podocyte cell line	Keir et al.	
Mouse IGF1R floxed podocyte cell line	This paper	

**Experimental models: Organisms/strains**		

podIGF1RKD mice	This paper	
pod2.IGF1RKD mice	This paper	

**Software and algorithms**		

ImageJ	NIH	https://imagej.nih.gov/ij/download.html RRID:SCR_003070
Leica Application Suite X software	Leica Microsystems	https://www.leica-microsystems.com/products/microscope-software/details/product/leica-las-x-ls/; RRID:SCR_013673
GraphPad Prism	GraphPad Software, San Diego, CA, USA	Version 9.4.0 RRID:SCR_002798
IN Cell Investigator	GE Healthcare	
Proteome Discoverer 2.1	Thermo Fisher Scientific	RRID:SCR_014477
Perseus software	MaxQuant	https://maxquant.net/perseus/ RRID:SCR_015753
STRING		http://string.embl.de/ RRID:SCR_005223

**Other**		

RPMI	Sigma-Aldrich	Cat# R8758
Fetal bovine serum	Sigma-Aldrich	Cat# F7524
VectaMount	Vector Laboratories	Cat# H-5000
DPX mount for histology	Sigma	Cat# 06522
Proteomic data	PRIDE partner repository	Dataset identifier PXD051018

### Resource availability

#### Lead contact

Further information and requests for resources should be directed to the lead contact Richard Coward [RJC] (Richard.Coward@bristol.ac.uk).

#### Materials availability

This study did not generate new unique reagents.

#### Data and code availability

##### Data availability

The mass spectrometry proteomics data is deposited on the ProteomeXchange Consortium platform via the PRIDE partner repository^61^ with the dataset identifier PXD051018.

### Experimental model and study participant details

#### Mouse models

Mice in which exon 3 of the IGF1R has been flanked by *loxP* sites^7^ were initially crossed with pCAGCre mice.^62^ Progeny with the genotype IGF1R^fl/null^ were subsequently crossed with podocin Cre mice to generate podocyte specific IGF1R knockdown animals from embryonic day 12 (podIGF1RKD). IGF1R^fl/null^ mice were crossed with pod2A Cre mice^22^ to generate pod2.IGF1RKO mice. Cre negative control littermates served as controls.

Doxorubicin nephropathy was induced in mice at 8–10 weeks of age by a single tail vein injection of doxorubicin (Merck) at 12 mg/kg body weight and urinary albumin:creatinine monitored for 2 weeks. Cre negative littermates served as controls.

All mice were on a mixed genetic background as we have used previously.^14^^,^^41^ Both sexes were studied, and no phenotypic differences observed. Transgenic mouse work was carried out in accordance with ARRIVE (Animal Research: Reporting of *In Vivo* Experiments) guidelines and procedures approved by the United Kingdom (UK) Home Office in accordance with UK legislation.

#### Cell lines

Mouse podocyte cell lines were cultured in Roswell Park Memorial Institute 1640 (RPMI-1640, Sigma-Aldrich Cat#R8758) media supplemented with 10% fetal bovine serum (FBS, Sigma-Aldrich Cat#F7524) at 33°C until 60–80% confluent, then allowed to differentiate for 10–14 days at 37°C as described previously.^63^^,^^64^

To generate the NC-IGF1RKD cell line kidneys were isolated from IGF1R^fl/fl^ mice (backcrossed with a membrane-targeted dimer Tomato/Cre mediated membrane-targeted Green Fluorescent Protein reporter mouse [mT/mG] such that GFP is expressed in the presence of Cre recombinase) and used to make a temperature-sensitive SV40 conditionally immortalised podocyte cell line as described previously.^63^ Cells were cultured in RPMI-1640 media supplemented with 10% FBS at 33°C and, when 50% confluent, were thermo-switched to 37°C and incubated for a further 7 days before transduction with a lentivirus expressing Cre recombinase as described previously.^41^ Transduction was in RPMI media with hexadimethrine bromide (Merck, Cat#H9268) at 4 μg/mL and the virus used at a multiplicity of infection of 1. Following a 24-h incubation, the lentivirus was removed and replaced with fresh media. Cells were incubated for a further 3–7 days before imaging and protein extraction.

### Method details

#### Urinary albumin and creatinine measurements

Albumin and creatinine levels in mouse urine were measured using spot collection in a mouse-specific albumin ELISA (Universal Biologicals, Cat# E90-134) and creatinine companion kit (Exocell, Cat# 1012), as described previously and following the manufacturers methodology.^41^

#### Periodic acid-schiff and Masson’s trichrome staining

Kidneys were fixed in 10% buffered neutral formalin (Merck, Cat# HT501128), further processed and paraffin embedded. Tissue sections of 3 μm were cut and stained using periodic acid-Schiff (Sigma, Cat#395B) and Masson’s trichrome (Sigma, Cat# HT15) staining kits according to the manufacturer’s instructions. Tissues were imaged using a Leica DN2000 microscope and micrographs taken using Leica Application Suite X software (Leica Microsystems; RRID:SCR_013673). Image analysis was performed using ImageJ (RRID:SCR_003070); all images were contrast enhanced using the same parameters.

Quantification was performed by measuring the area of glomerular PAS positive staining or blue coloration (indicating fibrosis in Masson’s trichrome staining) in ≥5 glomeruli in 3 mice per group.

#### Electron microscopy

Tissues for electron microscopy were fixed in 0.1 M sodium cacodylate, 2% glutaraldehyde, and imaged on a Technai 12 transmission electron microscope. Average slit diaphragm, foot process and glomerular basement membrane width were calculated using ImageJ (RRID:SCR_003070) assessing at least 20 regions of glomerular basement membrane from at least 2 glomeruli per mouse.

#### Immunofluorescence

Frozen kidneys were sectioned at 5 μm. Sections were blocked in phosphate buffered saline (PBS) containing 3% bovine serum albumin (BSA) and 0.3% Triton X-100 for 1 h, then incubated with primary antibodies overnight at 4°C (P-p44/42MAPK 1:100; nephrin 1:200). Following 3 phosphate buffered saline (PBS) rinses, sections were incubated with fluorophore-conjugated secondary antibodies (Fisher) for 1 h at room temperature. Tissues were imaged using a Leica DM2000 microscope and micrographs taken with Leica Application Suite X software (Leica Microsystems; RRID:SCR_013673). Image analysis was performed with ImageJ (RRID:SCR_003070); all images were contrast enhanced using the same parameters.

#### RNAscope *in situ* hybridisation

Tissues were fixed in 10% buffered neutral formalin, processed and paraffin embedded. Tissue sections of 3 μm were then prepared for RNAscope chromogenic single-plex probe analysis. Slides were baked in a dry oven for 1 h at 60°C before being deparaffinised with xylene twice for 5 min, followed by 100% ethanol twice for 1 min. Slides were air dried for 5 min at room temperature. Hydrogen peroxide was applied to each section and target retrieval performed by heating sections for 20 min in target retrieval reagent (Biotechne, Cat# 322000) at 98°C–100°C. Protease plus (Biotechne, Cat# 322330) was applied for 40 min at 40°C. Hybridisation was carried out at 40°C for 2 h using WT1 and IGF1R (targeting exon 3) probes (Biotechne, Cat# 417798 and Cat# 432711-C2) on serial sections. For WT1 detection, signal amplification and detection reagents (Biotechne, Cat# 322500) were applied sequentially and incubated in AMP 1, AMP 2, AMP 3, AMP 4, AMP 5, and AMP 6 reagents, for 30, 15, 30, 15, 40, 15 min respectively. For IGF1R detection, sections were subject to additional amplification steps with AMP 7, AMP 8, AMP 9 and AMP 10 reagents for 15, 30, 60 and 15 min respectively. Before adding each AMP reagent, samples were washed twice with washing buffer. The samples were then counterstained with 50% Gill’s haematoxylin I (Sigma, Cat# GHS132) for 2 min at room temperature, rinsed with tap water, followed by another tap water rinse. Samples were dried for 15 min in a 60°C dry oven. VectorMount mounting media (Vector Laboratories, Cat# H-5000) and cover slips were then added to slides before imaging using a Leica DN2000 microscope and Leica Application Suite software (Leica Microsystems; RRID:SCR_013673).

#### Insulin and IGF1 stimulation of podocytes

For acute insulin and IGF1 stimulation, Conditionally immortalised wild-type mouse podocyte cell were serum starved for 4 h then 10 nM and 100 nM of insulin (Biotechne, Cat# 3435) or 10 ng/mL and 100 ng/mL IGF1 (Novus, Cat# NBP2-35081) was applied to the cells for 10 min. Cells were treated with picropodophyllin (Stratech, Cat# S7668) at 100 nM for 24 h. For the doxorubicin injury model, cells were incubated with doxorubicin (Merck, Cat# D1515) at the concentrations indicated for 24 h.

#### Cell survival assay

To determine NC-IGF1RKD cell number, cells were washed 3 times in PBS, the nuclei stained with Hoechst (Thermo Fisher Scientific, Cat# H3570) at 1 μg/mL and imaged using an IN Cell analyser 2200 and data analyzed using IN Cell analyser Developer software (GE Healthcare).

#### Western blotting

Cultured cells were lysed in radioimmunoprecipitation assay buffer (RIPA; Thermofisher, Cat# 10017003) supplemented with protease and phosphatase inhibitors (Merck, Cat# P8340, Cat# P5726 and Cat# P0044). 10–30 μg of protein was resolved by electrophoresis and then transferred to a polyvinylidene difluoride membrane. Membranes were blocked in TRIS-buffered saline with 0.1% tween 20 and 5% BSA for 1 h and subsequently incubated overnight with primary antibody at a dilution of 1:1000. Membranes were washed before incubation with horseradish peroxidase conjugated secondary antibody (Sigma, Cat# A6667; RRID:AB_ 258307 and Cat# A9044; RRID:AB_ 258431). Immunoreactive bands were visualised using Clarity ECL Western Blotting Substrate (BioRad, Cat# 1705061) on a GE AI600 imager. Densitometry was performed using ImageJ software (RRID:SCR_003070).

#### Proteomic analysis

NC-IGF1RKD podocytes (3 days after Cre lentiviral transduction and before significant cell loss) and P-IGF1RKD (wild-type podocytes treated with 100 nM picropodophyllin for 24 h) were lysed in RIPA buffer and subjected to LC-MS/MS using isobaric TMT labeling as described previously.^65^^,^^66^ Wild-type mouse podocytes served as controls.

The initial data output generated from Proteome Discoverer 2.1 software (Thermo Fisher Scientific, RRID:SCR_014477) was further analyzed in Microsoft Office Excel, Graphpad Prism 9 (RRID:SCR_002798) and Perseus 1.6.10.43 (MaxQuant, RRID:SCR_015753) using log_2_-transformed scaled total protein abundance data. A *t*-test was used to determine significantly differentially expressed proteins. Hierarchical clustering was performed in Perseus by Euclidean distance using *k*-means pre-processing. Gene ontology (GO), Kyoto Encyclopedia of Genes and Genomes (KEGG) and Reactome enrichments were performed in Perseus using a Fisher test of significantly changed proteins versus the nonchanged population after annotation with the proteins. Two-dimensional GO, KEGG and REACTOME enrichment analysis was performed on the log_2_ ratios of NC-IGF1RKD/wild type versus P-IGF1RKD/wild type.

For STRING (Search Tool for the Retrieval of Interacting Genes/Proteins,RRID:SCR_005223) enrichment analysis, NC-IGF1RKD and P-IGF1RKD expression datasets were submitted to STRING with log_2_ fold change values associated with each protein. Functional enrichments were identified, and enrichment scores calculated based on aggregate fold change or Kolmogorov-Smirnov testing in STRING.^67^

#### Immunohistochemistry

Tissues were fixed in 10% buffered neutral formalin (Merck, Cat# HT501128), processed and paraffin embedded. 3 μm sections were deparaffinised in Histo-Clear II (National Diagnostics, Cat# HS-202) and rehydrated through a graded alcohol series. Antigen retrieval was in 10 mM citrate buffer, pH6 for by boiling for 10 min. Sections were quenched using 3% H_2_O_2_, followed by a blocking step in 3% normal goat serum for 30–45 min. Sections were incubated overnight at 4°C with an NDUFB8 antibody diluted 1:50. Sections were washed then incubated with SignalStain Boost detection reagent (Cell Signaling Technology, Cat# 8114) for 30 min at room temperature. SignalStain DAB substrate kit (Cell Signaling Technology, Cat# 8059) was applied for 1–2 min and the sections dehydrated and mounted in DPX (Sigma, Cat# 06522). Tissues were imaged using a Leica DM2000 microscope and micrographs taken with Leica Application Suite X software (Leica Microsystems; RRID:SCR_013673). Image analysis was performed with ImageJ; all images were contrast enhanced using the same parameters.

#### Mitochondrial bioenergetics

A Seahorse bioanalyser was used to assess changes in mitochondrial bioenergetics in NC-IGF1RKD podocytes (3 days after IGF1R gene knockout) by measuring oxygen consumption rate (OCR). NC-IGF1RKD and control cells were seeded onto XF culture microplates (Agilent Technologies, Cat# 103015–100) at 2 × 10^4^ cells/well and 3 technical replicates assayed per experiment. OCR was measured before and after the injection of 2 μM oligomycin, 1 μM FCCP, 0.5 μM rotenone and 0.5 μM antimycin (Agilent Technologies, Cat# 103025–100) and used to calculate mitochondrial respiratory parameters as follows:

Basal respiration: (OCR before addition of oligomycin)-(non-mitochondrial respiration [OCR after rotenone/antimycin addition])

Proton leak: (OCR after oligomycin addition)-(non-mitochondrial respiration).

ATP production: (OCR before oligomycin addition)-(OCR after oligomycin addition).

Maximal respiration: (OCR after FCCP addition)-(non-mitochondrial respiration).

Spare capacity: (Maximal respiration)-(Basal respiration).

### Quantification and statistical analysis

Statistical analysis was performed in Graphpad Prism 9 (RRID:SCR_002798). Statistical details for the experiments are provided in the figure legends. Unless otherwise stated data are represented as the mean +/− standard error of the mean. *p* < 0.05 was considered significant.

## Supplemental information

Document S1. Figures S1‒S10

Table S1. GO and KEGG terms enriched NC-IGF1RKD vs. wild-type podocytes, related to Figure 5B

Table S2. GO and KEGG terms enriched P-IGF1RKD vs. wild-type podocytes, related to Figure 5C
