# Supplementary material for: Contrasting consequences of podocyte insulin-like growth factor 1 receptor inhibition
Source: iScience. 2024 Apr 16;27(5):109749. doi: 10.1016/j.isci.2024.109749 (PMC11068853; doi:10.1016/j.isci.2024.109749)
Supplement: Document S1. Figures S1‒S10 [file mmc1.pdf]

iScience, Volume *27*

Supplemental information

Contrasting consequences of podocyte insulin-like growth factor 1 receptor inhibition

Jennifer A. Hurcombe, Fern Barrington, Micol Marchetti, Virginie M.S. Betin, Emily E. Bowen, Abigail C. Lay, Lan Ni, Lusyan Dayalan, Robert J.P. Pope, Paul T. Brinkkoetter, Martin Holzenberger, Gavin I. Welsh, and Richard J.M. Coward

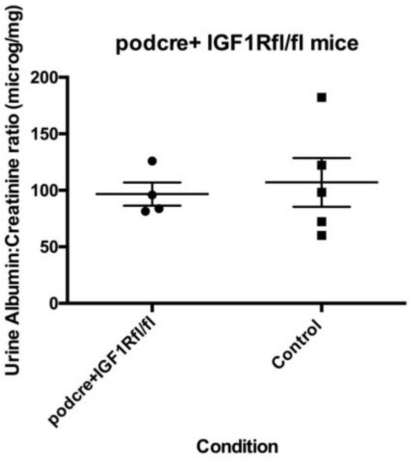

Figure S1. PodocinCre+IGF1R$^{fl/fl}$ mice studied at 4-5 months of life with age matched Cre negative controls. Data are expressed as the mean +/- SEM, n=4-5 per group.

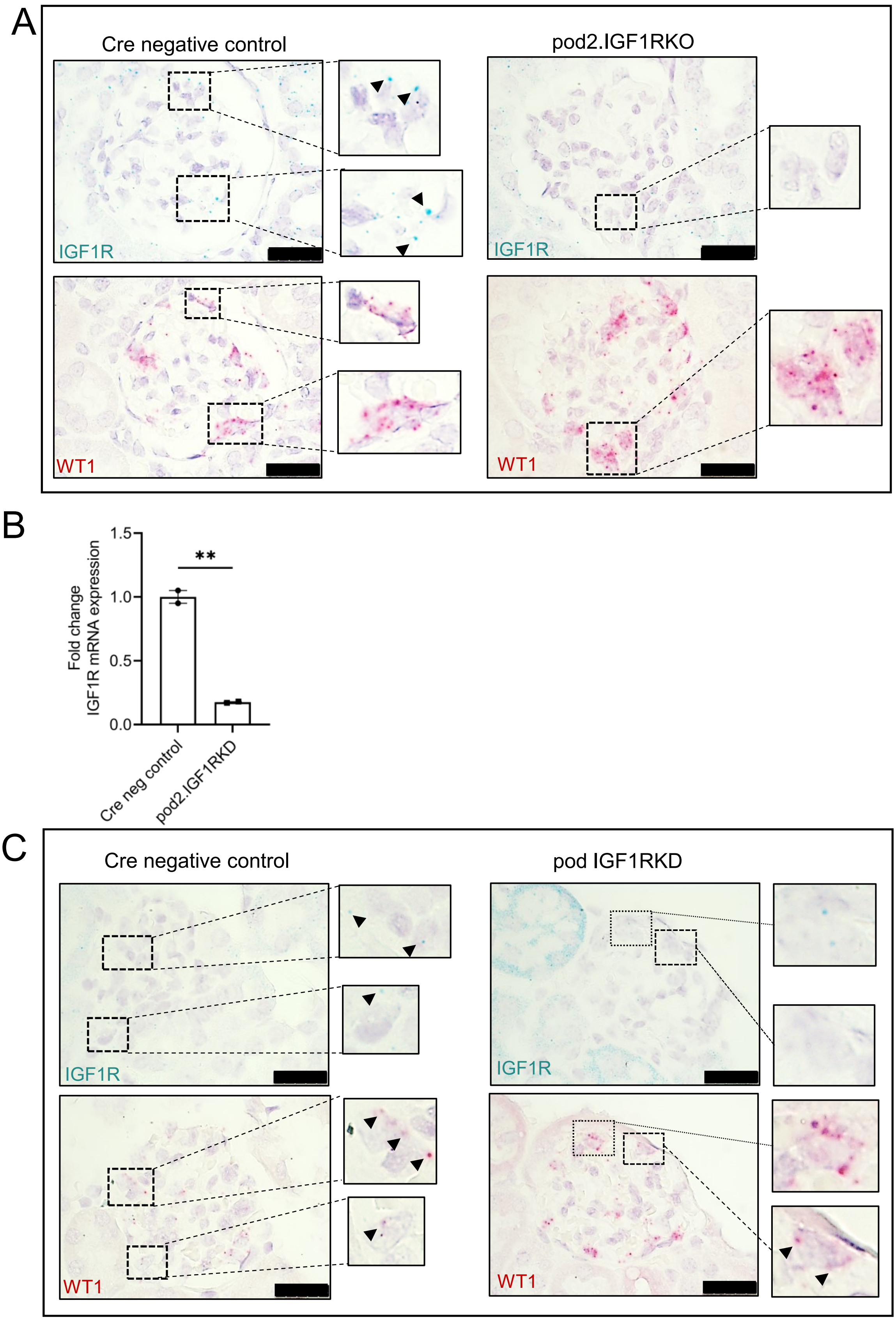

Figure S2. RNAscope *in situ* hybridisation to assess podocyte IGF1R expression in pod IGF1RKD and pod2.IGF1RKO mice. Related to Figures 1 and 2.

A Representative images showing IGF1R expression (green, indicated by arrowheads) in podocytes (WT1+ cells, red) of Cre negative control but not pod2.IGF1RKO mice. Scale bar=25 µm.

B Fold change in podocyte IGF1R mRNA expression. Quantification was performed by counting IGF1R transcripts in WT1 positive cells according to the following scoring criteria: 0= no staining; 1=1-3 dots/cell; 2=4-9 dots/cell; 3=10-15 dots/cell; 4=>15 dots/cells. 7-10 glomeruli per mouse were analysed and an average score per glomerulus calculated. Data are expressed as the mean +/- SEM, t test, **p<0.01.

C Representative images showing IGF1R expression (green, indicated by arrowheads) in podocytes (WT1+ cells, red, indicated by arrowheads) of Cre negative control mice. The weak signal is likely due to sub optimal fixation of historic podIGF1RKD samples. No IGF1R expression in most podocytes of podIGF1RKD mice (large dashes) though IGF1R transcripts can be detected in a number of WT1 positive cells (small dashes). Scale bar=25 µm.

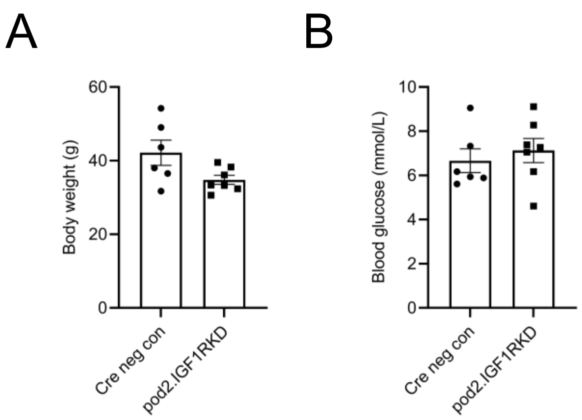

Figure S3. Body weight and blood glucose levels are unchanged in pod2.IGF1RKD mice at 6 months. Related to Figure 2.
A No difference in body weight between pod2.IGF1R KD and littermate controls at 6 months.
B No difference in blood glucose between pod2.IGF1RKD mice and littermate controls at 6 months. Data are expressed as the mean +/- SEM, 6-7 mice per group.

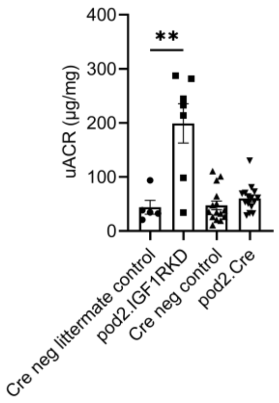

Figure S4. pod2.Cre mice do not develop albuminuria. Related to Figure 2.
No difference in uACR between Cre negative pod2.IGF1RKD littermate controls, Cre negative and pod2.Cre expressing mice at 6 months. uACR is significantly increased in pod2.IGF1RKD mice. t test, **p<0.01, n=5-15 mice per group.

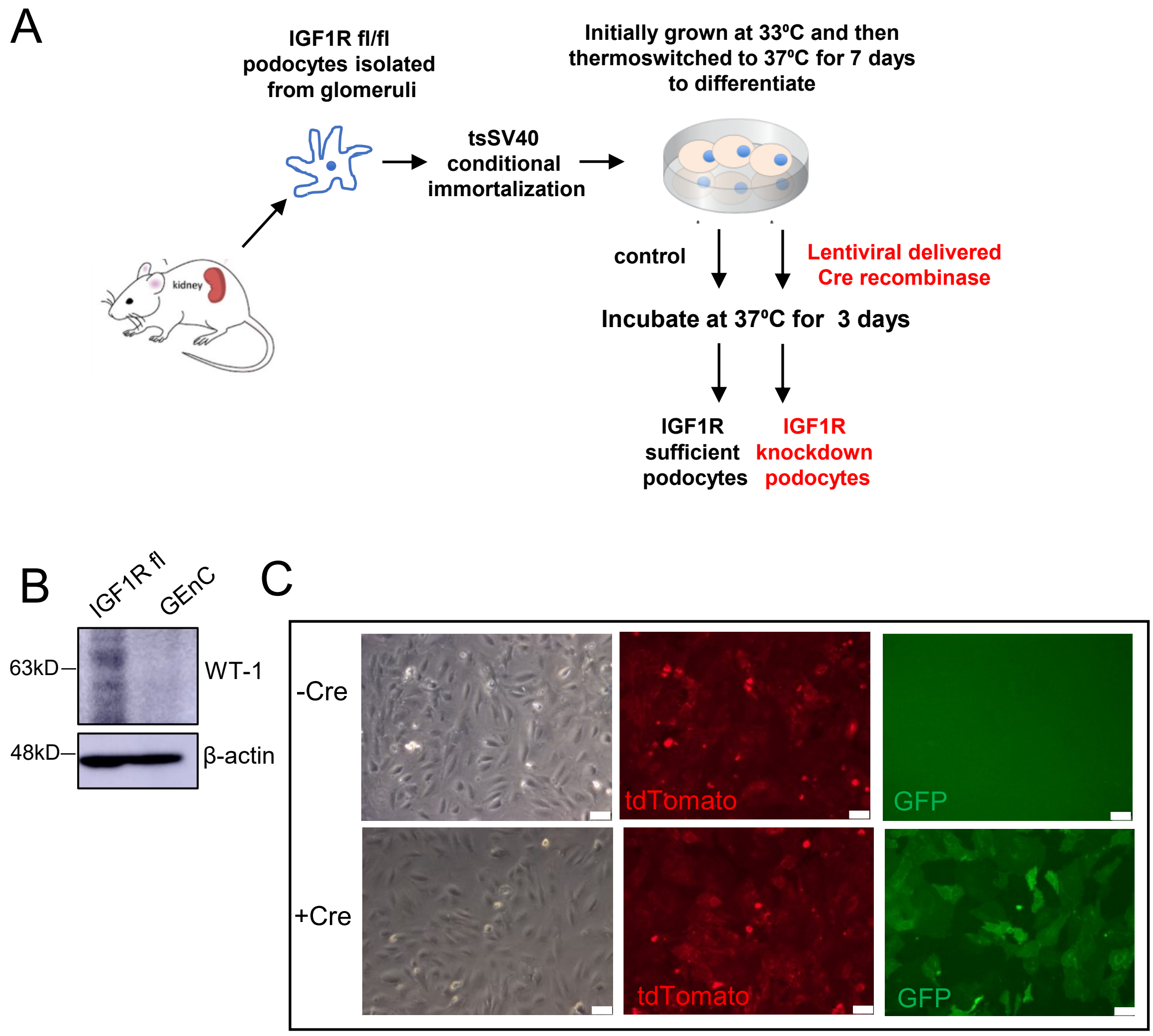

Figure S5. Development of NC-IGF1RKD conditionally immortalised podocytes. Related to Figure 3.

A Development of NC-IGF1RKD conditionally immortalised podocytes. Primary culture podocytes isolated from transgenic mouse. Conditional immortalisation with temperature sensitive SV40 construct and then excision of IGF1R using lentiviral delivered Cre recombinase.

B Western blot of IGF1Rfl cell lysate showing expression of the podocyte marker WT1. Human glomerular endothelial cells were used as a negative control.

C IGF1R fl podocytes harbouring a td tomato/GFP reporter gene (which expresses GFP in the presence of Cre recombinase) were imaged 3 days after transduction with a Cre expressing lentivirus. GFP expression is visible in transduced cells but not in the non-transduced control. Scale bar=50μm.

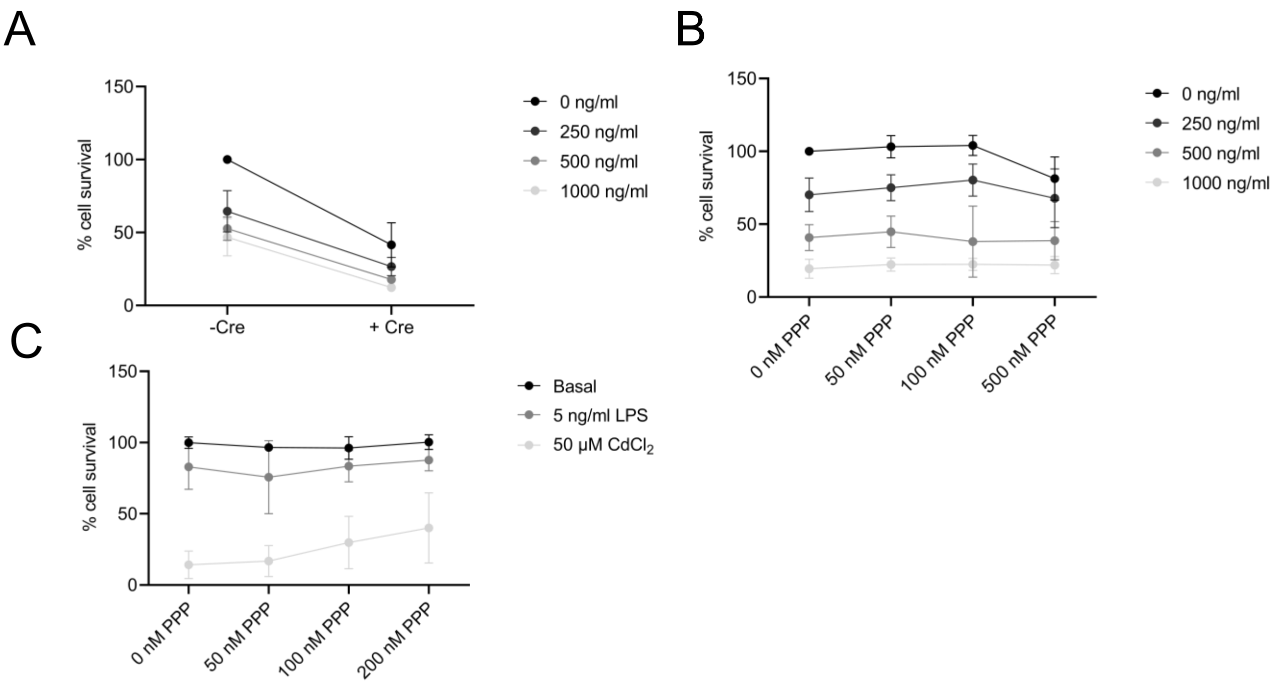

Figure S6. Preliminary dose response experiments to determine the effect of picropodophyllin (PPP) in *in vitro* podocyte injury models. Related to Figure 4.

A Cell survival of NC-IGF1RKD cells treated for 24 hours with doxorubicin 3 days after Cre lentiviral transduction.

B Preliminary dose response experiment to determine the optimal PPP dose to attenuate doxorubicin-induced podocyte death. Wild-type podocytes were incubated for 24 hours with doxorubicin at 0-1000 ng/ml and PPP at 0-500 nM before assessment of cell number. The pro-survival effect of PPP was optimal at 100 nM.

C Preliminary dose response experiment to determine the effect of PPP on cell survival in additional models of podocyte injury. Wild-type podocytes were incubated for 24 hours with LPS at 5 ng/ml or $CdCl_2$ at 50 μM and with PPP at 0-200 nM.

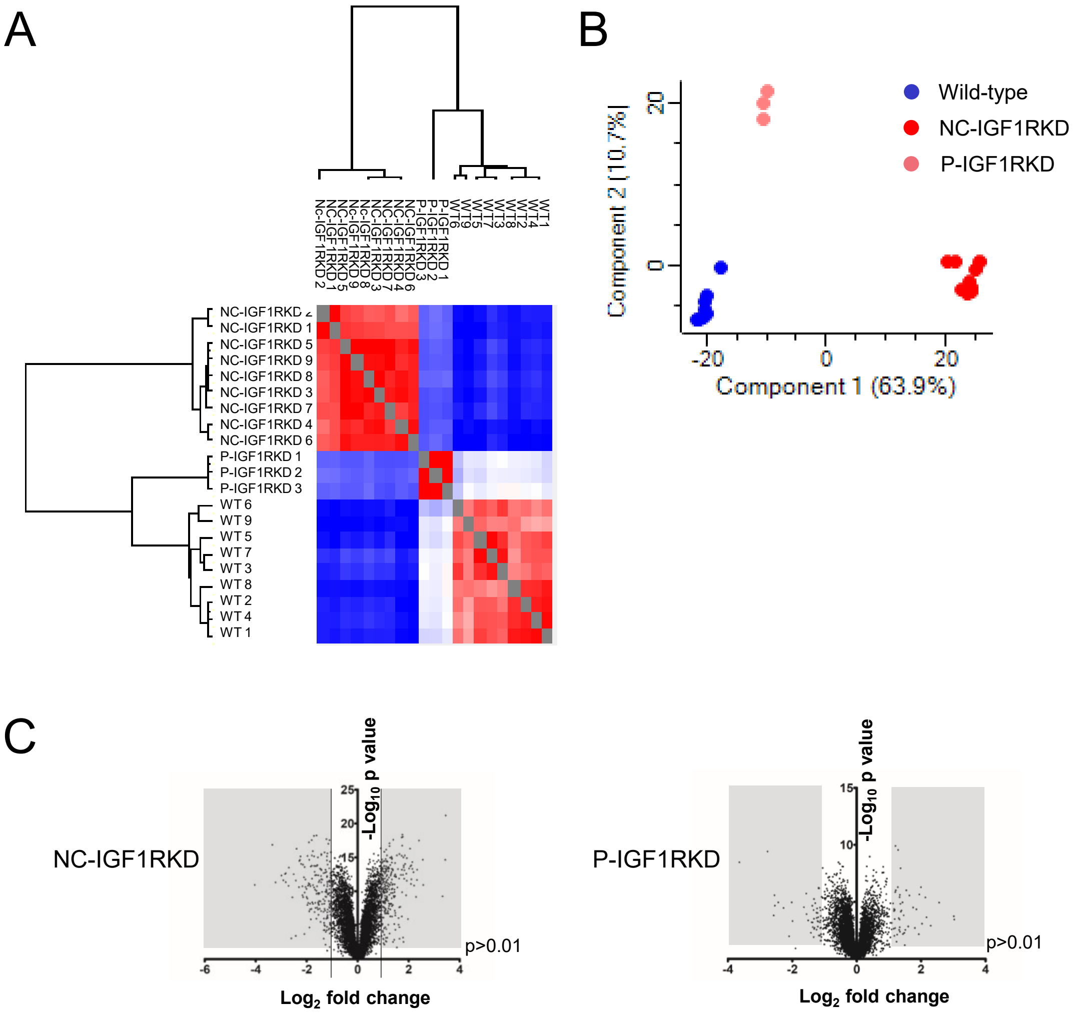

Figure S7 Proteomic analysis of NC-IGF1RKD and P-IGF1RKD podocytes. Related to Figure 5.
A Heat map of sample clustering
B Plot of principal component analysis
C Volcano plots showing changes in the proteome of NC-IGF1RKD and P-IGF1RKD podocytes relative to wild-type control cells; $\log_2$ fold change (FC) vs $-\log_{10}$ p-value of the scaled abundances. Shaded areas show proteins with FC < and > 2.

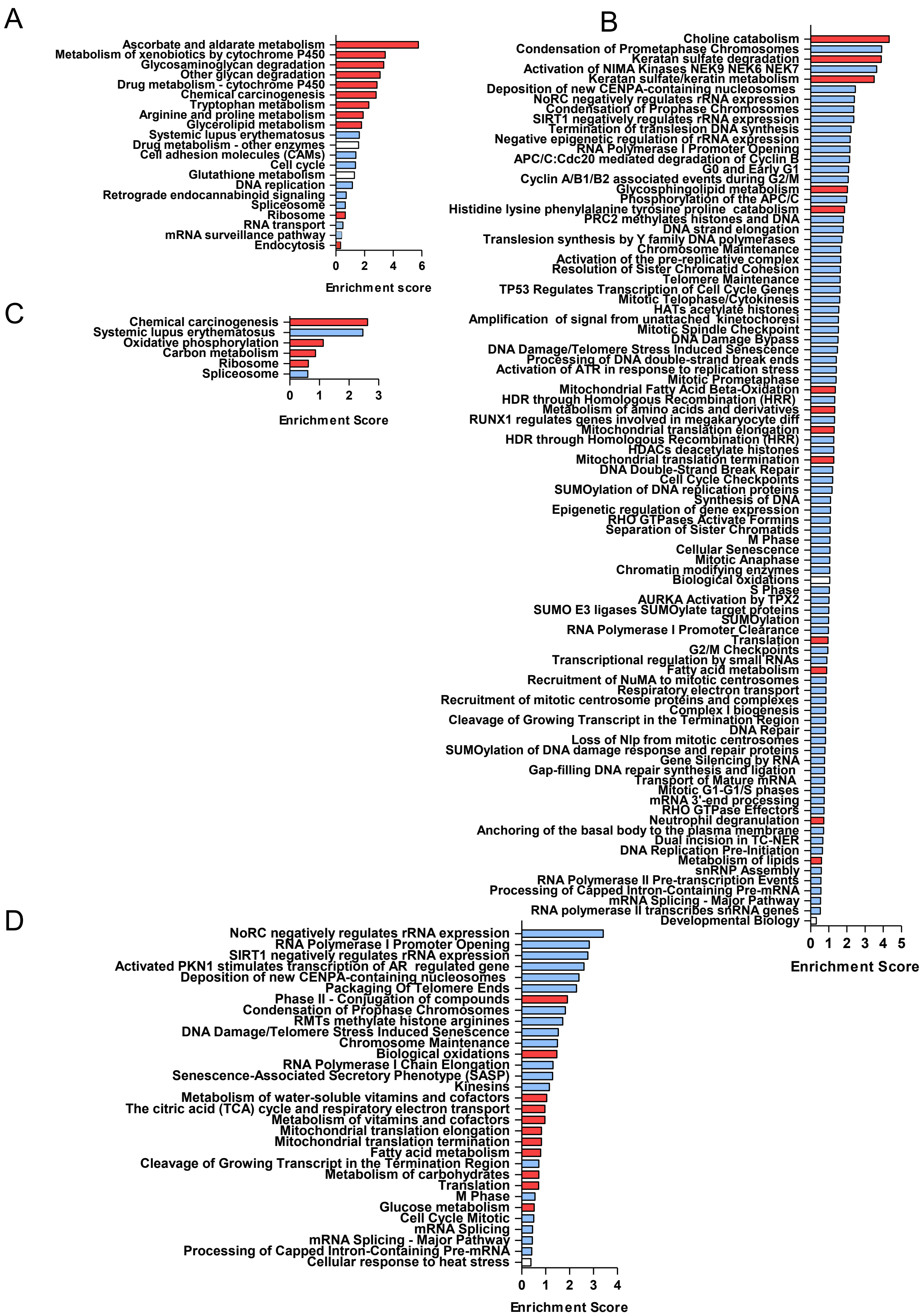

Figure S8. Enrichment analysis of NC-IGF1RKD and P-IGF1RKD podocyte proteomes performed in STRING. Related to Figure 5.

A and **B** KEGG pathways enriched in NC-IGF1RKD **(A)** and P-IGF1RKD podocytes **(B).**

C and D Reactome pathways enriched in NC-IGF1RKD **(C)** and P-IGF1RKD podocytes **(D)**

Blue:pathways associated with downregulated proteins; red:pathways associated with upregulated proteins; white:pathways associated with up and downregulated proteins.

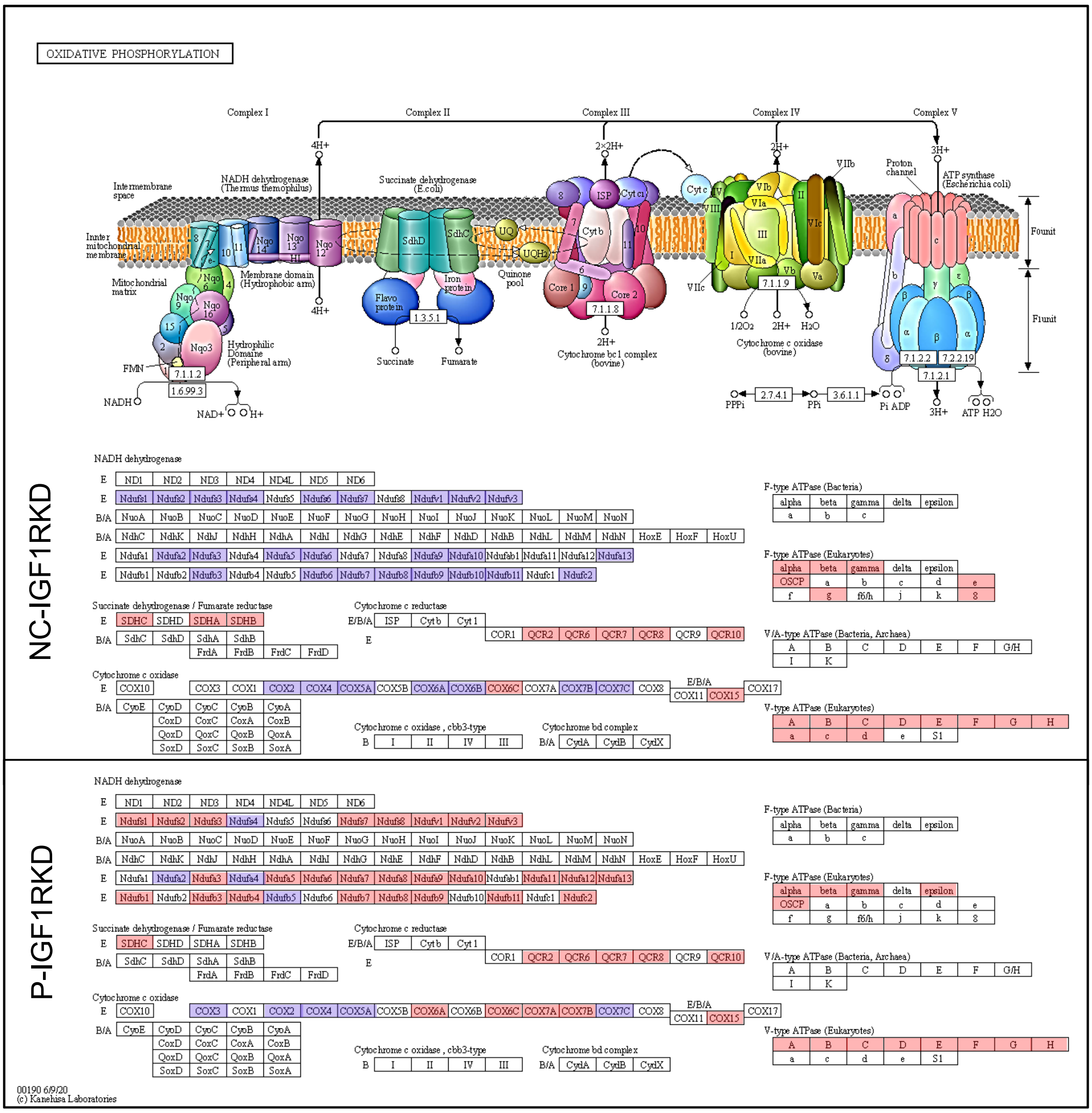

Figure S9. Differential expression of mitochondrial respiratory electron transport chain proteins in NC-IGF1RKD and P-IGF1RKD proteomes. Related to Figure 6.
Mitochondrial electron transport chain proteins upregulated (red) or downregulated (blue) in NC-IGF1RKD and P-IGF1RKD proteomes mapped onto the oxidative phosphorylation KEGG pathway shows reduced expression of respiratory complex I proteins in NC-IGF1RKD podocytes.

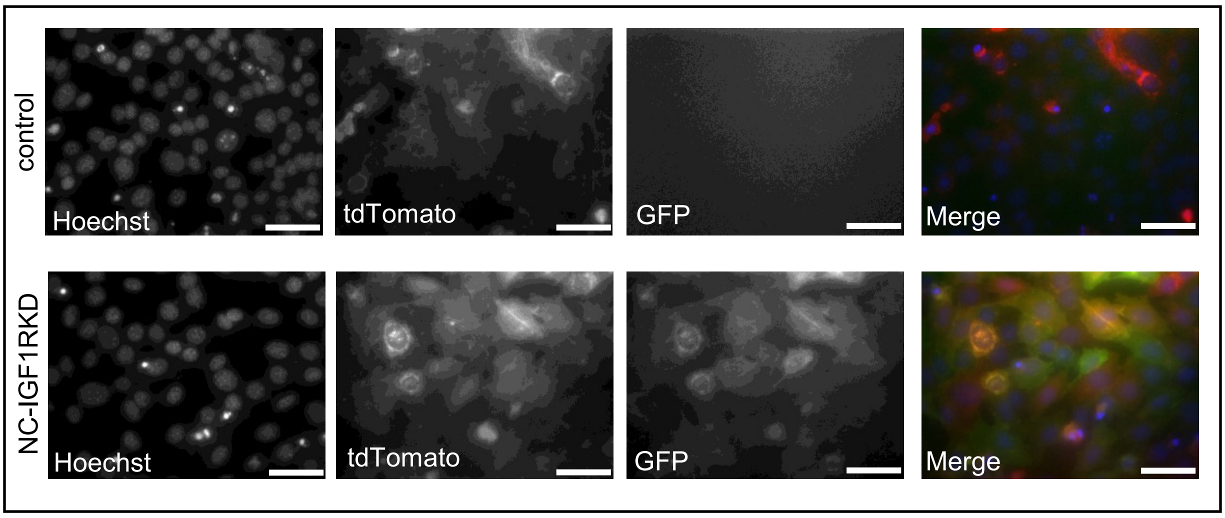

Figure S10. Images of NC-IGF1RKD and control cells used for Seahorse studies. Related to Figure 7.
GFP expression is present in NC-IGF1RKD cells indicating successful Cre-lentiviral transduction. Scale bar=500µm
